# Penile Cancer Distant Metastasis or Primary Lung Cancer? Using Focused Genomic Profiling of Tumor and Germline Mutations With Next‐Generation Sequencing for Clinical Decision‐Making

**DOI:** 10.1002/cnr2.70278

**Published:** 2025-07-15

**Authors:** Christian A. Moen, Ida M. Nordanger, Ása Karlsdóttir, Alfred Honoré, Patrick Juliebø‐Jones, Siri M. Blomberg, Torjan M. Haslerud, Christina Aamelfot, Pirjo‐Riitta Salminen, Christian Beisland, Hildegunn H. Vetti, Daniela E. Costea, Ellen Berget

**Affiliations:** ^1^ Department of Urology Haukeland University Hospital Bergen Norway; ^2^ Department of Clinical Medicine University of Bergen Bergen Norway; ^3^ Department of Oncology Haukeland University Hospital Bergen Norway; ^4^ Department of Radiology Haukeland University Hospital Bergen Norway; ^5^ Department of Radiology Nuclear Medicine, Stavanger University Hospital Stavanger Norway; ^6^ Department of Thoracic Medicine Haukeland University Hospital Bergen Norway; ^7^ Section of Cardiothoracic Surgery, Department of Heart Disease Haukeland University Hospital Bergen Norway; ^8^ Western Norway Familial Cancer Center, Haukeland University Hospital, Bergen, Norway and VID Specialized University, Faculty of Health Studies Bergen Norway; ^9^ Department of Pathology Haukeland University Hospital Bergen Norway

**Keywords:** *ERBB2* mutation, genomic profiling, metastasis, next generation sequencing, penile squamous cell carcinoma, *TP53* mutation

## Abstract

**Background:**

The presence of a distant metastasis in penile squamous cell carcinoma (PSCC) is associated with a very poor prognosis. When isolated distant tumors are detected in patients with known PSCC, it is therefore important to accurately determine whether such lesions represent penile cancer metastasis or indeed a new primary cancer. This distinction can have a significant influence on both patient prognostication as well as recommended treatment regimens.

**Cases:**

In this case series, we present three patients surgically treated for inguinal node‐positive PSCC. Two of the patients developed isolated lesions in the right hilar lymph nodes within 14 months after surgery, and one patient had a concurrent lesion in the right upper lobe at the time of diagnosis. None of the standard radiological or histopathological examinations could truly identify the origin of these lesions. Moreover, neither could biomarker analysis with p16^INK4a^ and human papillomavirus (HPV) DNA status. However, focused genomic profiling of both penile and thoracic tumor tissue with next generation sequencing (NGS) technology identified specific mutations in the *TP53* gene (2 cases) and a potentially actionable mutation in the *ERBB2* gene (1 case) that additionally could aid in distinguishing possible primary lung SCC from metastatic PSCC. No germline mutations were detected.

**Conclusion:**

Focused NGS analysis of tumor tissue can provide molecular insights that may help clarify the possible origin of thoracic tumors in patients with PSCC. The results may support clinical decision‐making and also be used for prognostication and patient counseling.

## 1 Introduction

The natural progression of penile squamous cell carcinoma (PSCC) has been described as an initial lymphatic spread to the inguinal and then to the pelvic lymph nodes [1]. Spread beyond this point has been defined as a distant metastasis and can involve the retroperitoneal/paraaortic and hilar glands as well as dissemination into visceral organs (notably the lungs) and bone [2, 3, 4].

Occasionally, isolated tumors involving hilar lymph nodes or lung tissue are discovered at initial work‐up or during follow‐up of PSCC. The question then arises whether such isolated tumors represent distant metastatic disease or indeed another primary malignancy. Accurate distinction between these two scenarios is important as it has a profound impact on patient prognosis as well as dictating the available and correct treatment options.

In this case series, we present three patients treated for inguinal node‐positive PSCC at Haukeland University Hospital in 2022/23. Two patients developed isolated lesions in the right hilar lymph nodes within 14 months after surgery, and one patient had a concurrent lesion in the right upper lobe at the time of diagnosis. Cases were discussed in multidisciplinary team meetings (MDT), and treatment was provided as per their discretion. However, ordinary radiological and histopathological examinations could not really disclose the origin of these lesions. Thus, after patient consent, we retrospectively analyzed the hospital records for clinical variables and disease course. The human papillomavirus (HPV) status of tumor tissue from all tumors was determined by the multiplex‐fluorescent HPV (f‐HPV) typing kit (Genomed LTD, London, United Kingdom) which can detect DNA from the following HPV types: 6, 11, 16, 18, 31, 33, 35, 39, 45, 51, 52, 56, 58, 59, 66, and 68. In addition, all tumors underwent p16^INK4a^ immunohistochemical staining using the JC2 clone (Cell Marque, California, USA). Furthermore, focused genomic profiling using the commercially available Oncomine Precision Assay (Thermo Fisher Scientific, Waltham, Massachusetts, USA) was performed in order to identify possible mutational signatures from both the penile and distant tumor. Currently, this next‐generation sequencing (NGS) assay enables detection of 2769 different variants of hotspot mutations, copy number variations, and gene fusions across 50 cancer genes (Table 1). This assay, however, does not provide tumor mutational burden (TMB) or microsatellite instability (MSI) status. To also investigate for possible germline mutations, blood samples were analyzed using the TruSight Hereditary Cancer Panel (Illumina, San Diego, California, USA), a commercially available NGS panel that assesses germline mutations across 113 genes known to be associated with hereditary cancer development.

**TABLE 1:** Oncomine precision assay gene list.

DNA hotspot mutations				
AKT1	CHEK2	FGFR3	KIT	NTRK3
AKT2	CTNNB1	FGFR4	KRAS	PDGFRA
AKT3	EGFR	FLT3	MAP2K1	PIK3CA
ALK	ERBB2	GNA11	MAP2K2	PTEN
AR	ERBB3	GNAQ	MET	RAF1
ARAF	ERBB4	GNAS	MTOR	RET
BRAF	ESR1	HRAS	NRAS	ROS1
CDK4	FGFR1	IDH1	NTRK1	SMO
CDKN2A	FGFR2	IDH2	NTRK2	TP53

CNVs		Gene fusions		
ALK	FGFR1	ALK	FGFR2	NTRK3
AR	FGFR2	AR	FGFR3	NUTM1
CD274	FGFR3	BRAF	MET	RET
CDKN2A	KRAS	EGFR	NRG1	ROS1
EGFR	MET	ESR1	NTRK1	RSPO2
ERBB2	PIK3CA	FGFR1	NTRK2	RSPO3
ERBB3	PTEN			

Abbreviation: CNV, copy number variations.

Based on clinicopathological characteristics as well as tumor and germline genetics, we then discuss the possible etiology of these thoracic lesions.

## 2 Cases

### 2.1 Case 1

**FIGURE 1:**
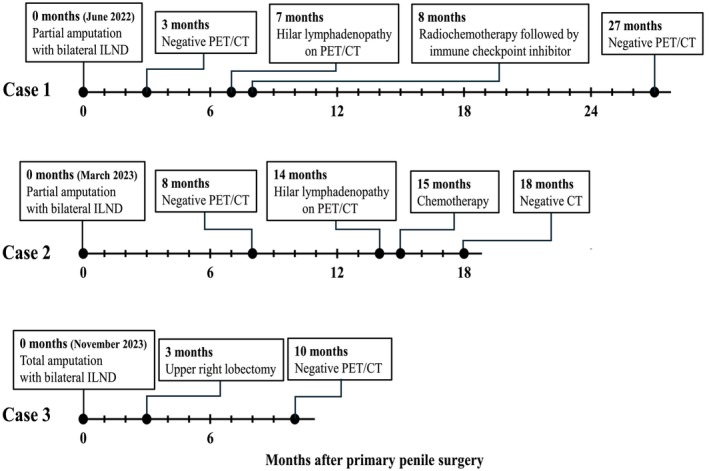
Timeline of main events for three different penile cancer patients with inguinal lymph node metastasis who either developed hilar lymphadenopathy during follow‐up (Case 1 and 2) or had a concurrent lung lesion in the upper right lobe at time of diagnosis (Case 3). ILND = Inguinal lymph node dissection; PET/CT = ^18^F‐fluoro‐2‐deoxy‐D‐glucose positron emission tomography combined with computed tomography.

**FIGURE 2:**
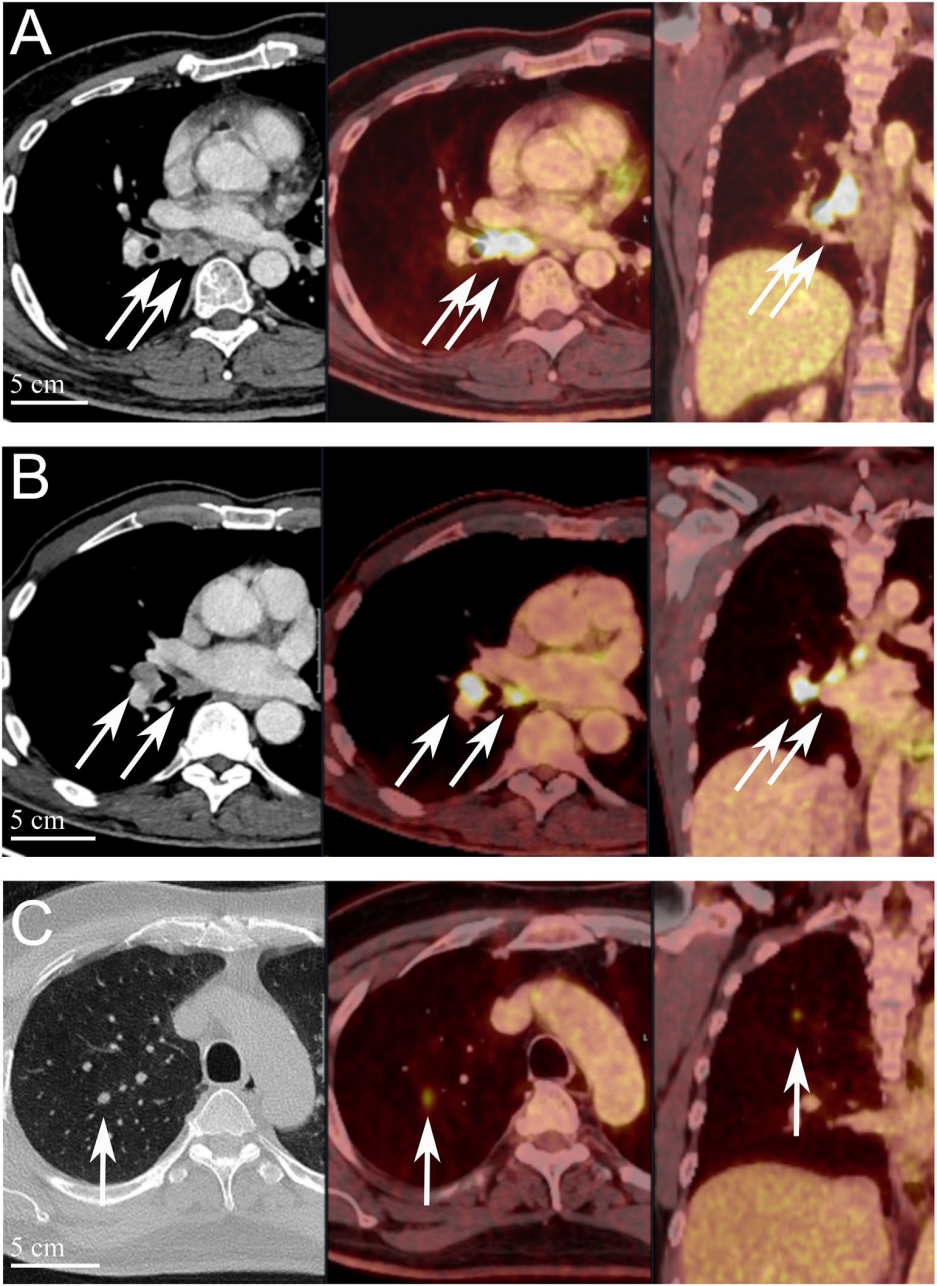
^18^F‐fluoro‐2‐deoxy‐D‐glucose positron emission tomography combined with computed tomography (FDG PET/CT) images of thoracic lesions in three different penile cancer patients. (A) FDG PET/CT positive lesion in right hilar lymph nodes (arrows) present 7 months after the patient was treated for an inguinal node‐positive penile cancer. (B) FDG PET/CT positive lesion in right hilar lymph nodes (arrows) present 14 months after the patient was treated for an inguinal node‐positive penile cancer. (C) Concurrent FDG PET/CT positive lesion in right upper lobe (arrows) of a patient that were simultaneously diagnosed and treated for an inguinal node‐positive penile cancer.

**FIGURE 3:**
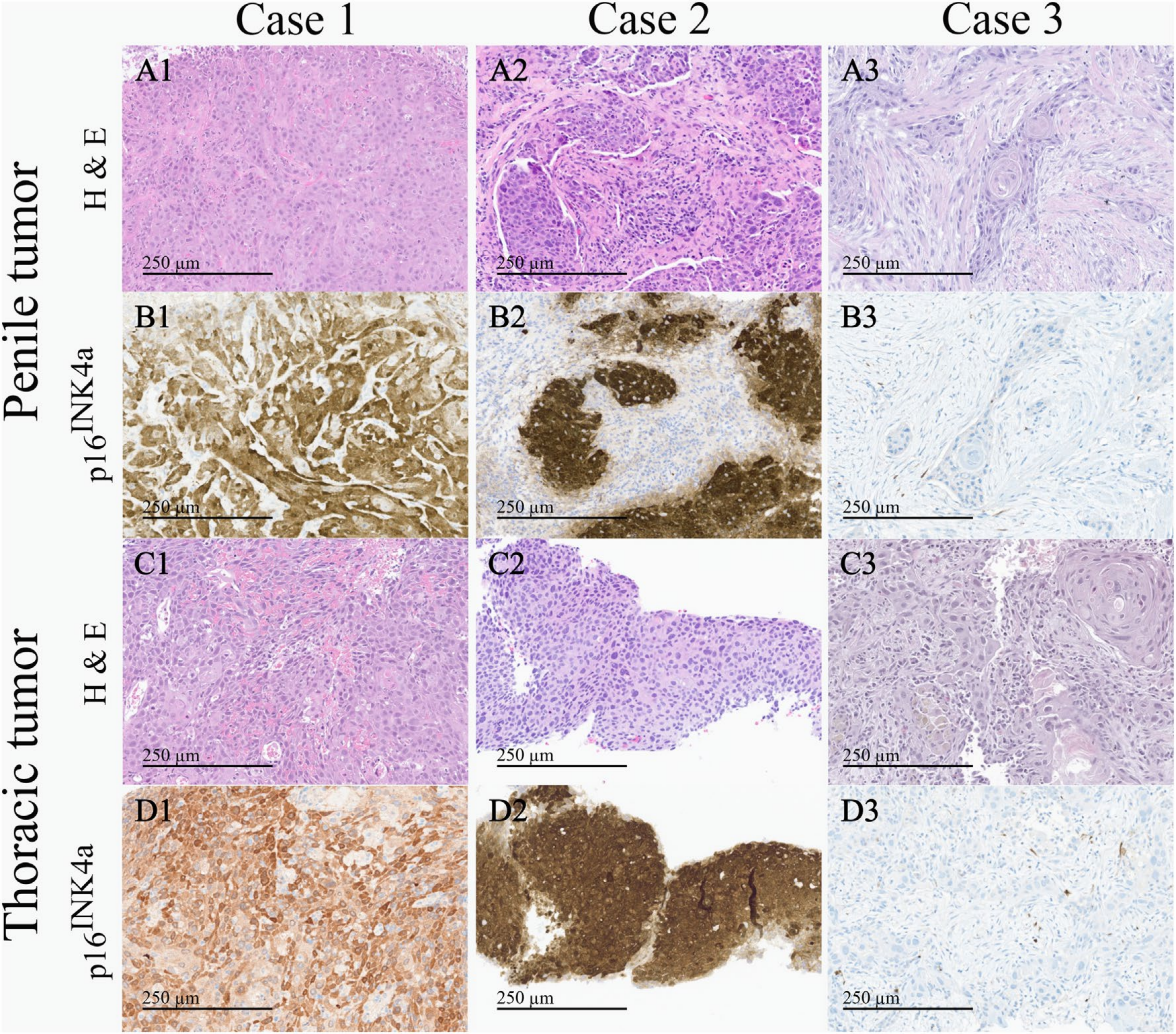
Histopathological images at 20X magnification (all images have a width of 662 μm and a height of 470 μm, with a scale bar of 250 μm shown in all panels). (A1—A3) Images showing hematoxylin and eosin (H & E) staining of penile tumor tissue from three patients with penile cancer. (B1—B3) Corresponding images of the penile tumor tissue after staining with p16^INK4a^. (C1—C3) Images showing H & E staining of thoracic tumor tissue from the same three patients with penile cancer. (D1—D3) Corresponding images of the thoracic tumor tissue after staining with p16^INK4a^.

**FIGURE 4:**
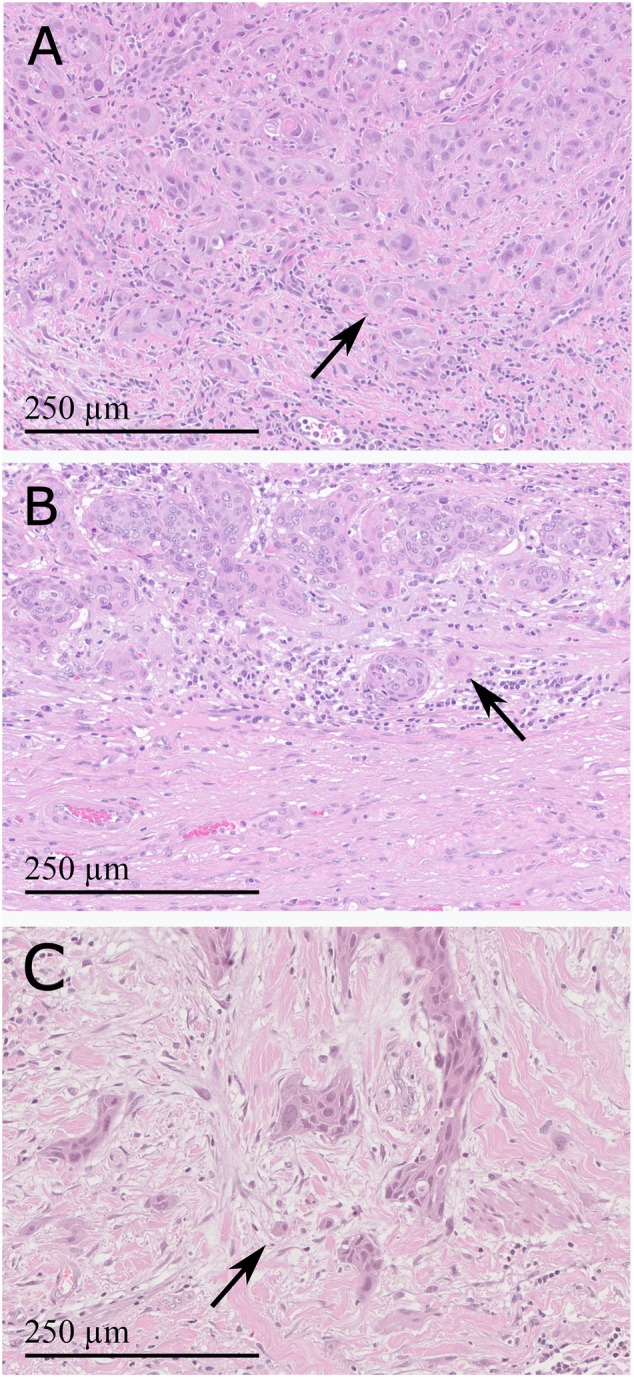
Histopathological images showing the tumor invasion front in three patients with inguinal lymph node‐positive penile cancer. Arrows indicate an example of tumor budding. (A) Patient (Case 1) with a *TP53* mutation detected in the tumor tissue, (B) Patient (Case 2) with an *ERBB2* mutation detected in the tumor tissue, (C) Patient (Case 3) with a *TP53* mutation detected in the tumor tissue. All images taken at 20X magnification (with a width of 662 μm and a height of 470 μm. A scale bar of 250 μm is shown in all panels).

In June 2022, a previously healthy 57‐year‐old man was referred to the Haukeland University Hospital for examination due to a painful genital ulcer at the coronal sulcus. Biopsy revealed PSCC. Inguinal nodes were not palpable (cN0). Whole‐body ^18^F‐fluoro‐2‐deoxy‐D‐glucose positron emission tomography combined with computed tomography (FDG PET/CT) was performed preoperatively. Except for FDG uptake in the penile tumor and inguinal lymph nodes bilaterally, there were no other signs of disease. The patient underwent a partial penectomy and bilateral inguinal lymph node dissection (ILND), which revealed metastasis to one node on the right side and two nodes on the left side. A timeline of the patient's main events is shown in Figure 1. Clinicopathological characteristics are summarized in Table 2. FDG PET/CT performed after 3 months did not show any sign of pathology. A new FDG PET/CT after 7 months, however, showed isolated right hilar lymphadenopathy with multiple enlarged nodes (Figure 2A). Endobronchial ultrasound (EBUS) bronchoscopy identified an enlarged lymph node with impression into the lower lobe bronchus where an irregular mucosal surface was identified. Biopsy from this area revealed SCC. In MDT meetings, based on radiological (Figure 2A) and histopathological findings (Figure 3, panels A1—D1 and Figure 4A), it was not possible to conclude on the origin of the tumor. The case was treated as a Stage 3 (locally advanced) primary non‐small cell lung cancer. The patient underwent concomitant radiochemotherapy (Cisplatin/vinorelbine) resulting in complete regression of the right hilar lymph nodes on FDG PET/CT. Since the tumor was PD‐L1 positive (tumor proportion score 99% on immunohistochemical staining with the PD‐L1 SP263 clone), he was then put on an immunotherapy regimen with an immune checkpoint inhibitor (durvalumab) once every 4 weeks for a year. However, this treatment had to be stopped after 4 months due to the development of pneumonitis, which was successfully treated with prednisolone. An FDG PET/CT performed 20 months after treatment of the thoracic lesions (27 months after surgery) did not show any residual disease. Currently, he is followed by surveillance with imaging every 6 months.

**TABLE 2:** Patient and tumor characteristics.

	Case 1	Case 2	Case 3
Patient characteristics			
Age (years)	57	68	55
Smoking status	Previously heavy smoker (30/day for 30 years)	Previously smoker (Periodically in his youth)	Previously heavy smoker (20/day for 35 years)
Previous cancer diagnosis	No	No	No
cN‐stage at primary surgery	cN0	cN0	cN0
Time to thoracic lesion discovery	7 months	14 months	Concurrent
Primary tumor and ILND characteristics			
Penile tumor size (mm)	3	14	55
Histological subtype	Usual SCC	Usual SCC	Usual SCC
pT‐stage	T1a	T2	T3
WHO grade	G2	G3	G2
pN‐stage	N2 (2 left, 1 right)	N2 (1 left, 1 right)	N1 (1 node left side)
LVI	No	Yes, extensive	No
Resection margin	R0	R0	R0
HPV DNA status	Negative	HPV‐16 DNA positive	Negative
p16^INK4a^	Positive	Positive	Negative
Thoracic tumor characteristics			
HPV DNA status	Negative	HPV‐16 DNA positive	Negative
p16^INK4a^	Positive	Positive	Negative

Abbreviations: cN, Clinical N‐stage; HPV, Human papillomavirus; ILND, Inguinal lymph node dissection; LVI, Lymphovascular invasion; pT‐stage, Pathological tumor stage; SCC, Squamous cell carcinoma; WHO, World health organization.

When investigated, the NGS analysis of tissue from the primary penile tumor and the right hilar lymph node biopsy revealed the same *TP53* R273H (Ex 8, p.(Arg273His)) mutation, known to be oncogenic due to loss‐of‐function, in both tumors (Table 3). This would support the possibility that the thoracic lesion represented distant metastatic PSCC.

**TABLE 3:** Comparison of the results of the genomic profiling in primary penile cancer tissue and isolated thoracic tumor lesions.

	Penile tumor mutations	Thoracic tumor mutations
Case 1	*TP53* R273H Mutation	*TP53* R273H Mutation
Case 2	*ERBB2* S310F Mutation	*ERBB2* S310F Mutation
Case 3	*TP53* R175H Mutation & *TP53* D184AfsTer62 Mutation	No mutation Detected With current NGS panel

Abbreviations: ERBB, Erythroblastic oncogene B; NGS, Next‐generation sequencing; TP, Tumor protein.

There were no germline mutations associated with cancer predisposition detected by the hereditary NGS cancer panel.

### 2.2 Case 2

In March 2023, a previously healthy 68‐year‐old man was referred to the Haukeland University Hospital for further investigation of an ulcer on the glans penis. There were no palpable inguinal nodes (cN0). A biopsy confirmed PSCC. A preoperative whole‐body FDG PET/CT revealed uptake in the penile tumor only. Partial amputation and ILND bilaterally were performed after intraoperative frozen section analysis of the dynamic sentinel node biopsies (DSNB) revealed node‐positive disease bilaterally. No additional inguinal lymph nodes contained cancerous tissue. A timeline of the patient's main events is shown in Figure 1. Clinicopathological characteristics are summarized in Table 2. An FDG PET/CT scan 8 months postoperatively was without any sign of recurrence. A new FDG PET/CT performed 14 months after surgery, however, showed right hilar lymphadenopathy with multiple enlarged nodes (Figure 2B). Biopsy by EBUS bronchoscopy confirmed SCC. It was again not possible to accurately assess the origin of the tumor. The histopathological findings are shown in Figure 3 (panels A2—D2) and Figure 4B. This specific case was, however, interpreted to be consistent with a distant PSCC metastasis. The patient underwent chemotherapy with a TIP regimen (cisplatin/paclitaxel/ifosfamide). He responded well to the treatment and is currently under active surveillance with imaging every 3–6 months.

Additional investigation confirmed the presence of HPV 16 DNA also in the hilar lymph node biopsy. Furthermore, NGS analysis revealed the same *ERBB2* S310F (Ex 8, p.(Ser310Phe)) mutation, known to be oncogenic due to gain‐of‐function, in the penile and the distant tumor (Table 3). This supports the notion that the hilar lesion was likely distant metastatic PSCC.

No germline mutations associated with predisposition for cancer development were detected by the current NGS panel.

### 2.3 Case 3

In November 2023, a previously healthy 55‐year‐old man was referred to the Haukeland University Hospital due to a large tumor covering most of the penis. Inguinal palpation was normal (cN0). A biopsy confirmed PSCC. A preoperative FDG PET/CT revealed uptake in the penile tumor without any other sign of locoregional pathology. However, an isolated small FDG PET positive lesion was observed in the lung tissue of the right upper lobe (Figure 2C). During PSCC surgery a total penectomy was performed. Bilateral DSNB with peroperative frozen sectioning revealed a micrometastasis (1.1 mm) on the left side but a normal lymph node on the right side. However, due to a suspect lesion of the skin in the right groin (which turned out to be benign), ILND was performed bilaterally. No other nodes contained cancerous tissue. A timeline of the patient's main events is shown in Figure 1. Clinicopathological details are shown in Table 2. Following recovery of the penile cancer surgery, biopsy of the lung lesion confirmed SCC. Again, no ordinary radiological or histopathological (Figure 3, panels A3—D3 and Figure 4C) parameter could disclose the origin of the tumor. The patient underwent a formal right upper lobe lobectomy with lymph node sampling. Histopathology described an SCC without any positive nodes. It was concluded that if this was primary lung cancer it would be staged as T1bN0. He would then already have undergone necessary treatment. Currently, therefore, the patient is only followed by active surveillance with imaging every 6 months. He has fully recovered, and an FDG PET/CT performed 10 months after PSCC surgery was without any sign of recurrence.

NGS analysis of tissue from the primary penile tumor revealed two mutations of the *TP53* gene. One mutation was *TP53* R175H (ex 5, p.(Arg175His)) which is known to be oncogenic due to loss‐of‐function. The other mutation, *TP53* D184AfsTer62 (Ex 5, p.(Asp184AlafsTer62)), is a truncating mutation that is likely oncogenic due to loss‐of‐function. NGS analysis of the SCC tumor of the lung tissue, however, did not reveal any mutations using the current NGS panel (Table 3). This would support the possibility that this patient in fact had two different primary tumors (lung and penis) at the time of PSCC diagnosis.

No germline mutations associated with cancer predisposition were detected by the hereditary NGS cancer panel.

## 3 Discussion

This case series investigated whether isolated thoracic tumors in patients with known PSCC represented distant metastasis or another primary cancer. A major challenge in the clinical setting was that the standard clinicopathological and radiological assessments were insufficient to determine the origin of the thoracic lesions. As a result, this led to differing interpretations and clinical management for each case, highlighting the need for additional diagnostic tools to accurately determine the origin of such lesions. The most significant finding of this study is the added value of performing focused NGS mutational analysis of both penile and distant tumors. This can produce additional evidence regarding the possible origin of distant lesions, thereby informing clinical decision‐making and supporting more appropriate and standardized recommendations on further treatment regimens. It also offers valuable insights that can be used for prognostication and patient information.

In a publication by Leijte et al., all distant PSCC metastases occurred within 16 months of primary treatment [5]. Moreover, in a study by Chakiryan et al., the median time to development of a distant lung recurrence was 9 months, and this was also associated with a worse overall survival compared to any of the other distant metastasis sites [6]. This highlights the importance of being able to confirm the origin of new thoracic lesions in patients with known PSCC.

Penile cancer guidelines recommend evaluation of both HPV and p16^INK4a^ status in penile cancer tissue [1]. Typically, HPV‐positive tumors are also p16^INK4a^‐positive, while HPV‐negative tumors are p16^INK4a^‐negative. The discrepancy in Case 1 (HPV‐negative, p16^INK4a^‐positive) remains unexplained, but it cannot be ruled out that this may be due to the presence of a more rare HPV type not included in the testing panel. Importantly, however, these biomarkers were consistent between the penile and distant tumors for all cases. If clinical decision‐making was to be based on these findings alone, the lung lesion in Case 3 would have been defined as metastatic PSCC. However, primary lung SCC can be both HPV and p16^INK4a^ positive [7]. Therefore, while discrepancies in these biomarkers could support different origins, similar findings cannot be used to conclude on the tumor origins.

Both patients with HPV negative penile cancer (Cases 1 and 3) had a *TP53* mutation. *TP53* mutations are known to be quite common in HPV negative, but rare in HPV positive, PSCC [8, 9, 10, 11]. Additionally, as for Case 1, high PD‐L1 expression was linked to HPV negativity in a recent study by Hrudka and colleagues [12]. In line with the advanced cases presented here, *TP53* mutations have been associated with an aggressive form of PSCC which often shows a high tumor budding count at the invasive front [13, 14, 15]. Morphologically, tumor budding was observed at the invasive front of all three penile tumors in this study, but was most pronounced for the two cases with a *TP53* mutation (Figure 4). Notably, the *TP53* R273H mutation found in Case 1 has previously also been described in a case of head and neck squamous cell carcinoma (HNSCC) with development of hilar lymphadenopathy and a lung nodule [16].

The *ERBB2* S310F mutation found in Case 2 is well known from cancers of the skin, urinary tract, and cervix [17]. Moreover, *ERBB2* mutations have also been described in penile cancer and have been associated with aggressive disease [10, 18, 19]. Interestingly, *ERBB2* mutations may represent actionable alterations and could serve as potential targets for future personalized treatment options [10, 19]. For penile cancer, however, there exists a broad spectrum of potentially druggable gene mutations, and the limited scope of our focused NGS panel precluded a comprehensive assessment of all of these relevant mutations [11, 20].

While the presence of identical mutations in both the primary tumor and the distant lesion can support a diagnosis of metastasis, it remains possible for common cancer‐associated mutations to arise independently in each tumor. Additionally, due to mutational heterogeneity and the ongoing evolution of mutations as cancer cells proliferate, primary and metastatic tumors may exhibit overlapping yet distinct mutation profiles. Thus, while focused NGS analysis can provide important information for clinical decision‐making, results must still be interpreted in the context of other case‐specific results. In this setting, more comprehensive analyses such as whole‐genome or whole‐exome sequencing could also have provided additional information.

In a study by Necchi et al., it was estimated that about 7% of the genetic alterations detected in penile cancer tumor tissue were germline in origin [18]. While we did not find germline mutations in any of these three cases with advanced and aggressive PSCC, more research into the role of germline mutations in penile cancer should be performed.

Using NGS analysis for detection of potentially actionable mutations as well as measurement of circulating tumor DNA (ctDNA) for disease monitoring may provide a future personalized treatment strategy in PSCC [21]. NGS analysis, however, offers the possibility of detecting many different mutations that again could form the basis of numerous different treatment regimens. Including these patients in dedicated trials should therefore be considered [22]. Moreover, outcomes of these rare cancer cases should be registered in large databases to be able to reasonably understand which treatment regimens are truly effective within this vast mutational landscape.

Assuming the cancer had not spread to the hilar lymph nodes at the time of initial treatment, one could speculate if a prophylactic pelvic lymphadenectomy (pPLND) could have prevented such development. According to current guidelines, however, significant controversy remains regarding the role of pPLND, and based on current literature, this has not presently been recommended for the patients in this study [1]. Possibly, with more studies on the association between tumor genetics and disease course, genomic profiling of tumors could help in the risk stratification regarding which patients should undergo pPLND in the future.

## 4 Conclusion

Focused NGS analysis of tumor tissue can provide information that can help determine the possible origin of thoracic tumors in patients with penile cancer. The results can be used to inform clinical decision‐making and may lead to a more standardized recommendation on further treatment regimens, while also offering information that can be used for prognostication and patient counselling.

## Author Contributions

**Christian A. Moen:** conceptualization, data curation, formal analysis, investigation, methodology, project administration, resources, software, supervision, validation, visualization, writing – original draft, writing – review and editing. **Ida M. Nordanger:** data curation, formal analysis, investigation, methodology, validation, writing – review and editing. **Ása Karlsdóttir:** data curation, formal analysis, investigation, writing – review and editing. **Alfred Honoré:** data curation, formal analysis, investigation, writing – review and editing. **Patrick Juliebø‐Jones:** data curation, formal analysis, investigation, validation, writing – review and editing. **Siri M. Blomberg:** data curation, formal analysis, investigation, software, validation, visualization, writing – review and editing. **Torjan M. Haslerud:** data curation, formal analysis, investigation, software, validation, visualization, writing – review and editing. **Christina Aamelfot:** data curation, formal analysis, investigation, validation, writing – review and editing. **Pirjo‐Riitta Salminen:** data curation, formal analysis, investigation, validation, writing – review and editing. **Christian Beisland:** data curation, formal analysis, investigation, validation, writing – review and editing. **Hildegunn H. Vetti:** data curation, formal analysis, investigation, methodology, validation, writing – review and editing. **Daniela E. Costea:** conceptualization, data curation, formal analysis, investigation, methodology, validation, visualization, writing – review and editing. **Ellen Berget:** conceptualization, data curation, formal analysis, investigation, methodology, software, supervision, validation, visualization, writing – review and editing.

## Ethics Statement

In accordance with local regulations, ethical board approval was not required for this type of research, but written informed consent was received from the patients for the publication of case details and use of images.

## Conflicts of Interest

The authors declare no conflicts of interest.

## Data Availability

The data that support the findings of this study are available on request from the corresponding author. The data are not publicly available due to privacy or ethical restrictions.
